# Identification of a Predicted Trimeric Autotransporter Adhesin Required for Biofilm Formation of *Burkholderia pseudomallei*

**DOI:** 10.1371/journal.pone.0079461

**Published:** 2013-11-05

**Authors:** Natalie R. Lazar Adler, Rachel E. Dean, Richard J. Saint, Mark P. Stevens, Joann L. Prior, Timothy P. Atkins, Edouard E. Galyov

**Affiliations:** 1 Department of Infection, Immunity and Inflammation, University of Leicester, Leicester, United Kingdom; 2 Biomedical Sciences, Defence Science and Technology Laboratory, Porton Down, United Kingdom; 3 The Roslin Institute and Royal (Dick) School of Veterinary Studies, University of Edinburgh, Easter Bush, United Kingdom; 4 School of Biosciences, Geoffrey Pope Building, University of Exeter, Exeter, United Kingdom; Tulane University School of Medicine, United States of America

## Abstract

The autotransporters are a large and diverse family of bacterial secreted and outer membrane proteins, which are present in many Gram-negative bacterial pathogens and play a role in numerous environmental and virulence-associated interactions. As part of a larger systematic study on the autotransporters of *Burkholderia pseudomallei*, the causative agent of the severe tropical disease melioidosis, we have constructed an insertion mutant in the *bpss1439* gene encoding an unstudied predicted trimeric autotransporter adhesin. The *bpss1439* mutant demonstrated a significant reduction in biofilm formation at 48 hours in comparison to its parent 10276 wild-type strain. This phenotype was complemented to wild-type levels by the introduction of a full-length copy of the *bpss1439* gene *in trans*. Examination of the wild-type and *bpss1439* mutant strains under biofilm-inducing conditions by microscopy after 48 hours confirmed that the *bpss1439* mutant produced less biofilm compared to wild-type. Additionally, it was observed that this phenotype was due to low levels of bacterial adhesion to the abiotic surface as well as reduced microcolony formation. In a murine melioidosis model, the *bpss1439* mutant strain demonstrated a moderate attenuation for virulence compared to the wild-type strain. This attenuation was abrogated by *in trans* complementation, suggesting that *bpss1439* plays a subtle role in the pathogenesis of *B. pseudomallei*. Taken together, these studies indicate that BPSS1439 is a novel predicted autotransporter involved in biofilm formation of *B. pseudomallei*; hence, this factor was named BbfA, *Burkholderia* biofilm factor A.

## Introduction

*Burkholderia pseudomallei* is a motile, Gram-negative bacillus, which is principally an environmental saprophyte of tropical and sub-tropical soils and water. It is the causative agent of melioidosis, a febrile illness with disease states ranging from acute pneumonia or septicaemia to chronic or localised abscess formation. The disease is acquired by subcutaneous inoculation, inhalation and ingestion. Melioidosis has become an increasingly important disease in endemic areas such as South East Asia and Northern Australia causing a significant number of deaths despite antibiotic treatment. *B. pseudomallei* has a large genome encoding many virulence factors, including capsule, lipopolysaccharide (LPS), flagella, quorum sensing (QS) systems and several protein secretion systems, including type III and type VI pathways [reviewed in [1], [2]].

*B. pseudomallei* grows in microcolonies *in vitro* and *in vivo* for which the production of exopolysaccharride have been implicated [3]. It has been postulated that biofilm formation may allow bacterial cells to evade the host immune system and persist within patients causing chronic or relapsing disease. Melioidosis is refractory to antibiotic therapy and *B. pseudomallei* biofilms have been shown to have very high levels of resistance to antibiotics which are effective against planktonic cells [4], [5]. The quantity of biofilm produced by *B. pseudomallei* is strain dependent [4], [6], [7] and affected by the growth conditions. Interestingly, the addition of glucose (2–50 mM) enhances biofilm formation [6]; the authors proposed that this may in part explain why chronic and relapsing infections are more common in diabetic patients with poor glycemic control [8].

The relative effects of a variety of mutants on the formation of biofilms have been tested. An acapsular *wcbB* mutant demonstrated wild-type levels of biofilm formation [4] while a second acapsular *wzm* mutant had reduced biofilm formation in minimal media but not in LB [9]. This may indicate that under certain conditions, capsule is important for biofilm production; however, the *wzm* mutant also demonstrated susceptibility to desiccation and oxidative stress [9] which may indirectly affect biofilm formation. An LPS O-antigen *wbiI* mutant also displayed wild-type levels of biofilm which concurs with the observation that the level of biofilm production by a strain does not correlate with the presence of O-antigen observed by silver staining [4].

An aflagellate *fliC* mutant demonstrated reduced biofilm formation [4]. Additionally, a *pkk* (polyphosphate kinase) mutant was found to be aflagellate and also demonstrated reduced biofilm formation; however, this mutant also displayed reduced sensitivity to oxidative stress [10]. To overcome the loss of flagella-based motility, centrifugation of these two mutants onto an abiotic surface was performed, but biofilm formation was still significantly reduced. Microscopy demonstrated that no microcolony formation occurred for either strain while the *pkk* mutant also lacked exopolysaccharide production [10]. These data indicate that flagella play a role in microcolony formation. However, this aflagellate phenotype can also be compensated by overexpression of other factors which promote microcolony formation. A *cpdA* mutant (encoding a c-di-GMP phosphodiesterase), with increased intracellular GMP levels, lacks flagella but produced increased levels of exopoolysaccharide, thus, more autoagglutination and biofilm formation [11].

It is unclear from the multiple phenotypes of the *cpdA* mutant whether c-di-GMP levels directly or indirectly affect biofilm. Intracellular levels of c-di-GMP affect the QS system [11]. *B. pseudomallei* has three QS synthases and five QS regulators [2]. A *bspI* mutant, which cannot produce the major stationary phase QS molecule C8HL, displayed reduced biofilm formation, as does a mutant in its regulator *bspR*. When the secondary producer of C8HL (*bspI3*) was inactivated, it also demonstrated reduced biofilm formation although comparatively less than the *bspI* mutant [12]. Biofilm levels could be restored to wild-type levels by the addition of exogenous C8HL [12]; these data indicated that one or more factors required for biofilm formation is/are expressed under stationary phase conditions and that this expression may be either directly or indirectly influenced by the QS regulator(s). Another regulatory factor that has been identified to potentially play a role in biofilm formation is the alternative sigma factor σ^E^ [13]. Proteomic analysis of protein expression by a *B. pseudomallei rpoE* mutant relative to its parent strain did not identify any proteins with known or putative roles in biofilm production [14].

To date, only one study has focused on genes directly involved in biofilm formation as the mutations discussed above have pleiotropic effects. A random mutagenesis study identified two strains which produced very low levels of biofilm; the first mutant, M6, had a transposon insertion within a gene encoding a putative polysaccharide biosynthesis protein, and the second, M10, had an insertion in gene encoding a putative sugar transferase [7]. These mutants demonstrated wild-type virulence in the BALB/c melioidosis model [7] and, although demonstrating some reduction in antibiotic diffusion and viability, still show biofilm-like levels of antibiotic resistance [7], [15]. However, these unexpected results are likely to be caused by the mutagenesis system used in this study; a subsequent paper by the same authors' identified a non-specific reduction in biofilm production due to the transposon used [16]. Therefore, factors directly involved in biofilm formation, and the role of biofilm in virulence, remain to be elucidated.

The autotransporters (ATs) are a large and diverse family of bacterial secreted and outer membrane proteins, which are present in many Gram-negative bacterial pathogens and play roles in numerous environmental and virulence-associated interactions including persistence, adhesion, serum resistance and biofilm formation. ATs utilise the Type V secretion mechanism, one of seven recognised secretion pathways in Gram-negative bacteria. A typical AT will have a 20–400 kDa passenger domain that contains the effector functions and a 10–30 kDa β domain facilitating translocation across the outer membrane [17]. ATs can be further divided into classical ATs and trimeric autotransported adhesins (TAAs). TAAs are so named because each protein trimerises to form a single 12-stranded β barrel from their three 4-stranded β barrel domains. The functional passenger domain of a typical AT acts predominantly as an adhesin [17], [18]. The *B. pseudomallei* K96243 genome contains eleven predicted ATs, based on sequence similarity to known ATs [19], of which nine are predicted to be TAAs [20]. Only two TAA adhesins have been characterised; mutants in *boaA* and *boaB* are impaired in epithelial adhesion while expression of recombinant BoaA and BoaB in a heterologous *E. coli* host increased the adhesive phenotype of the bacterial cells [21]. As part of a larger systematic study on the autotransporters of *B. pseudomallei*, we have constructed an insertion mutant in the *bpss1439* gene encoding an unstudied predicted TAA. This mutant was found to be affected in its ability to form biofilm, and demonstrated a moderate attenuation for virulence compared to the wild-type strain in the acute murine melioidosis model, suggesting that production of biofilm may play a role in virulence of *B. pseudomallei*.

## Materials and Methods

### Ethics Statement

All investigations involving animals were carried out according to the requirements of the Animal (Scientific Procedures) Act 1986. Ethical approval was granted by our local (Defence Science and Technology Laboratory, University of Leicester) ethical review process according to the requirements of the Animal (Scientific Procedures) Act 1986. Predetermined humane end points were employed where possible and animals were culled via cervical dislocation according to schedule 1 of the Animal (Scientific Procedures) Act 1986.

### Bacterial strains, plasmids, media and growth conditions

All bacterial strains and plasmids reported in these studies are listed in Table 1%. The strains were routinely grown in Luria-Bertani (LB) broth with shaking or on 1.5 w/v agar at 37°C. Cultures were supplemented with antibiotics and/or 10 mM isopropyl-β-D-thiogalactopyranoside (IPTG) as required; kanamycin was used at a final concentration of 500 µg/ml and tetracycline at 25 µg/ml.

**Table 1:** Strains, plasmids and cell lines used in this study.

Strain, plasmid or cell line	Characteristics	Reference or source
*B. pseudomallei*:		
10276	Clinical isolate (wild-type)	Dr T. Pitt (Public Health Laboratory Service, London, UK)
10276 (pME)	10276 harbouring the pME6032 plasmid	This study
*bpss1439* (*bbfA*) mutant	10276 harbouring a pKNOCK-Kan single cross-over insertion in *bpss1439 (bbfA)*	This study
*bpss1439* (*bbfA*) mutant (pME)	10276 harbouring a pKNOCK-Kan single cross-over insertion in *bpss1439 (bbfA)* and the pME6032 plasmid	This study
*bpss1439* (*bbfA*) mutant (pME-*1439*)	10276 harbouring a pKNOCK-Kan single cross-over insertion in *bpss1439 (bbfA)* and the pME6032 plasmid containing a full-length copy of *bpss1439*	This study
*E. coli*:		
S17-1 λ*pir*	Laboratory strain (λ*pir hsdR pro thi*; chromosomal integrated RP4-2 Tc::Mu Km::Tn*7*).	[36]
Plasmids:		
pKNOCK-Kan	2.2 kb, Kan^R^, *λpir*-dependent vector (*ori*R6K) with RP4 *oriT*	[23]
pKNOCK-Kan-*bpss1439*	pKNOCK-Kan containing a 623 bp internal fragment of *bpss1439 (bbfA)*	This study
pME (pME6032)	9.8 kb, Tet^R^, pVS1 derived shuttle vector with IPTG inducible ptac promoter	[24]
pME-*1439*	pME6032 containing the full-length (4593 bp) *bpss1439 (bbfA)* gene, including 146 bp upstream	This study
*Eukaryotic cell lines:*		
J774.2	Murine macrophage-like cells	ECACC 85011428
A549	Human lung airway epithelial cells	ECACC 86012804

### Recombinant DNA techniques

Plasmid DNA isolation, PCR fragment purification and gel extraction were performed using Bioline kits as per manufacturer's instructions. Genomic DNA was isolated from *B. pseudomallei* as previously described [22]. Restriction endonucleases and DNA modifying enzymes (New England Biolabs) were used as per manufacturer's instructions. PCR amplifications were performed using Advantage Taq (Clontech) or Taq DNA polymerase (New England Biolabs) in accordance with manufacturer's instructions. DNA sequencing was carried out at the Protein and Nucleic Acid Laboratory of the University of Leicester.

The *bpss1439* single cross-over insertion mutant was generated using pKNOCK-Kan [23] as described previously. Briefly, an internal fragment of the *bpss1439* gene was amplified by PCR from *B. pseudomallei* 10276 using primers EF (5′ TCCGAGGGATCCAACGGCAAGCGCGGCGGC) and M5R (5′ CCCGTCTCTAGAGAAGTGGAAAGCGAGGTCAG). The PCR product containing the internal fragment was digested with *Bam*HI and *Xba*I alongside the pKNOCK-Kan plasmid and ligated to form pKNOCK-Kan-*bpss1439*. This recombinant plasmid was introduced into *B. pseudomallei* 10276 via conjugation from *E. coli* S17-1λ*pir* [23] and insertion inactivation of *bpss1439* was confirmed by PCR and sequencing.

To allow for *in trans* complementation, the full-length *bpss1439* gene was amplified by PCR from *B. pseudomallei* 10276 using primers FC (5′ ATCTCTAGACCATGATGAACAAGATCTATAAAACC) and RC (5′ ATCGGATCCTTCATGTGCTTGAGCCAGTC). The PCR product containing the *bpss1439* gene was digested with *Nco*I and *Bam*HI and ligated into similarly digested pME6032 [24] under the control of a *lac* promoter to form pME6032-*bpss1439* (pME-*1439*).

### Biofilm assays

Biofilm formation on an abiotic polystyrene surface was studied using 96-well trays (Nunc) as described previously [10]. Briefly, overnight cultures of *B. pseudomallei* strains were diluted 1∶10 in LB and incubated at 37°C under static conditions for 48 h. Biofilms were washed in distilled water, stained with 1% w/v crystal violet for 5 minutes, washed and then solubilised in 96% v/v ethanol. The degree of biofilm formation stained by the crystal violet was determined by the optical density at 595 nm. Eight experimental replicates were assessed in triplicate experiments.

### Microscopic analysis of biofilms

Biofilms were allowed to form on 20 mm glass coverslips (Menzel Gläser) within 24 well trays (Nunc) as described above and fixed overnight in 4% w/v paraformaldehyde or 2.5% v/v glutaldehyde for examination by light microscopy or scanning electron microscopy (SEM) respectively. For light microscopy, fixed biofilms grown on coverslips were visualised with a Peroidic Acid Scheiff stain (Sigma) and examined using a Nikon Diaphot 300 inverted microscope with a ×100 oil immersion lens. For SEM, fixed biofilm grown on coverslips were dehydrated, sputter coated with gold and examined using a Hitachi S3000H SEM (Leicester Imaging Technologies, University of Leicester). At least ten fields of view were assessed in duplicate experiments.

### Phenotypic testing

The *bpss1439* mutant and its parent wild-type 10276 strain were assessed for various phenotypes which may impact of biofilm formation using published methods. Phenotypes examined were: polysaccharide capsule using mAb staining [25], LPS using silver stain [4], flagella and pili using motility plates [10], oxidative stress using peroxide sensitivity [13], osmotic stress using NaCl sensitivity [13], desiccation using drying sensitivity [9] and exopolysaccharide production using Congo Red binding [11]. Also *in vitro* attachment and net intracellular survival assays were performed using immortalised A549 human airway epithelial cells [11] and J774.2 murine macrophage-like cells [13].

### Assessment of virulence in mice

Three independent animal trials to assess the virulence of the *bpss1439* mutant were performed as follows.

Groups of six BALB/c mice (6–8 weeks old) were challenged by the intra-peritoneal route with either the wild-type or *bpss1439* mutant at 3.5×10^5^ CFU or 3.07×10^5^ CFU. Mice were monitored for signs of disease over a 35 day period and humanely culled when pre-defined end-points were reached; a log-rank (Mantel-Cox) test was used to evaluate the statistical significance of time to death between the wild-type and *bpss1439* mutant.

Six groups of five BALB/c mice (6–8 weeks old) were separately challenged by the intra-peritoneal route with the *bpss1439* mutant at doses rising in ten-fold increments from 8.8×10^2^ to 8.8×10^7^ CFU. The wild-type 10276 parent strain was tested at a single dose (3×10^6^ CFU) to confirm consistent results with the previously determined MLD of 1.6×10^4^ CFU from 2004 and 2009 (data not shown). Mice were monitored for signs of disease over a 35 day period and humanely culled when pre-defined end-points were reached. The medium lethal dose for the *bpss1439* mutant was determined using the Reed and Meunch calculation based on cumulative infections.

Groups of three BALB/c mice (6–8 weeks old) were challenged by the intra-peritoneal route with an equal ratio of wild-type harbouring the pME6032 vector and the *bpss1439* mutant harbouring either the pME6032 vector or the pME-*1439* complementation vector (4×10^5^ CFU). Mice were monitored for signs of disease over the 24 hour experimental period and then humanely culled. Livers and spleens were harvested and plated for bacterial recovery. Colonies were patched onto kanamycin and tetracycline agar plates and the percentage of *in vivo* growth of the *bpss1439* mutant versus the wild-type was determined by subtracting the number of tet^R^, kan^R^ (*bpss1439* mutant harbouring pME6032 or pME-*1439*) colonies from the number of tet^R^, kan^S^ (wild-type harbouring pME6032) colonies. Statistical significance was assessed using Student's T test.

## Results and Discussion

### Bioinformatic analysis of BPSS1439, an unstudied predicted TAA

**Figure 1:**
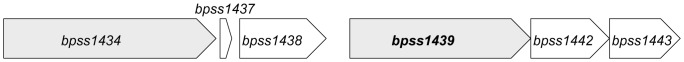
The arrangement of the *bpss1439* operon. The *bpss1439* gene is located in an operon with two downstream genes, *bpss1442* and *bpss1443*; both which encode hypothetical proteins of no significant database hits. The closest orthologue to *bpss1439* is the upstream gene (*bpss1434*) encoding another unstudied predicted TAA.

The *B. pseudomallei* K96243 genome encodes nine predicted TAAs; the *bpss1439* gene is located on chromosome two and has been predicted to be located in an operon with two downstream genes, *bpss1442* and *bpss1443* (C. Ong, personal communication; [26]) (Figure 1). Both of these downstream genes are annotated as encoding hypothetical proteins which lack homology to known proteins in available databases. As a result of revision of the K96243 genome annotation, there are no longer genes numbered *bpss1440* or *bpss1441*. The closest orthologue to *bpss1439* is the upstream gene *bpss1434*;, which is predicted to encode another unstudied TAA *bpss1434* is a significantly larger gene of 7899 bp (versus the 4581 bp *bpss1439*). These genes share 85% identity with most of the variation occurring in the 5′ end of the gene which encodes the functional N-terminal region of the predicted TAA (data not shown) suggestive that these genes encode TAAs with divergent functions. The high degree of sequence similarity between *bpss1439* and *bpss1434* has resulted in the mistaken earlier observation that orthologues of both these genes are present in *B. thailandensis* E264 [27], which in fact only has a full-length orthologue for *bpss1434* and a truncated pseudogene (639 bp) in place of *bpss1439*. Interestingly, the *bpss1439* orthologue also occurs as a pseudogene in *B. mallei* ATCC 23344 (BMAA0810; 459 bp). Furthermore, truncation of *bpss1439* also occurs within some of the sequenced *B. pseudomallei* strains (14, 112, 91, NCTCC13177, BCC215, 7894, DM98, B7210, BPC006, 1106b, 668); these pseudogenes not only vary in length but in whether they are 5′ or 3′ truncations. Other strains have a full-length copy of the *bpss1439* gene (MSHR346, K96243, 1106a, 1710a, 1710b, MSHR305, 1026b) with small size differences due to variable numbers of repeat regions (1488 bp to 1616 bp). Agarose gel electrophoration of the PCR amplification of the *bpss1439* gene from the 10276 strain used in this study indicated that a full-length copy was present on the genome (data not shown).

The *bpss1439* gene encodes for a 1530 aa predicted TAA which contains an extended signal peptide (pfam13018) with a predicted cleavage site at 24 aa, and the characteristic C-terminal domains annotated as HIM (pfam05662; short motif found in invasins and haemoagglutinins) and YadA-like (pfam03895; beta barrel region) [28]. The remainder of the protein largely consists of low-complexity repeat regions which form the neck structure responsible for ensuring the adhesin domains are accessible to their host target. A single cross-over insertion mutation disrupting the *bpss1439* gene was constructed using the sucide vector pKNOCK-Kan in which a 623 bp internal fragment of *bpss1439* was cloned. The resulting plasmid was recombined into *bpss1439*; sequencing of an amplicon of the mutated region on the chromosome confirmed the disruption of the gene at a point which would produce a transcript encoding a severely truncated 207 aa protein lacking both a portion of the passenger domain and the C-terminal domain responsible for mediating outer member translocation (data not shown).

### The bpss1439 mutant displayed reduced biofilm formation compared to the wild-type strain

**Figure 2:**
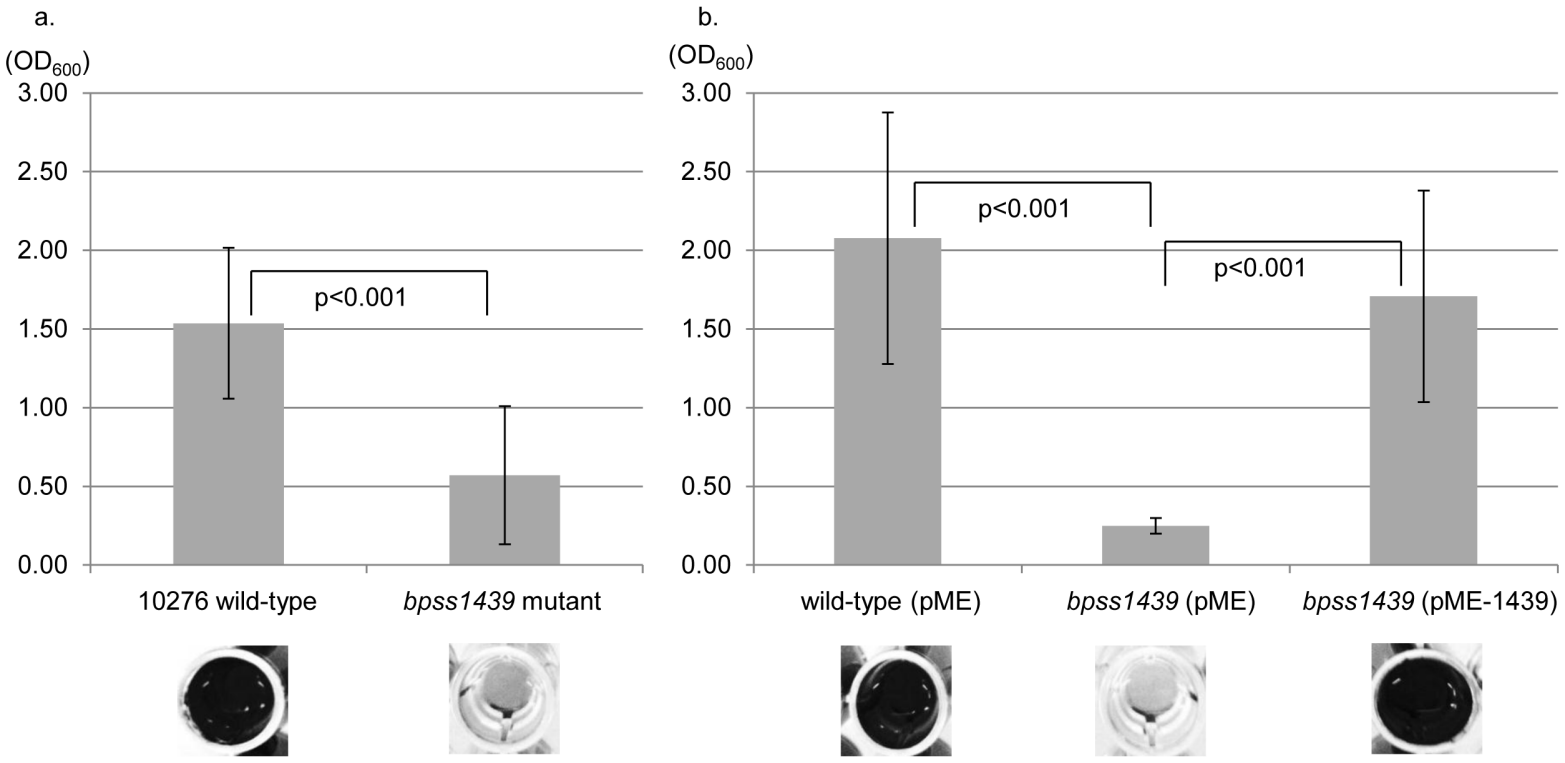
The *bpss1439* mutant displayed reduced biofilm formation which was complemented by *in trans bpss1439* expression. a. The wild-type and *bpss1439* mutant were grown in LB under static conditions at 37°C for 48 hours. Biofilms were stained with 1% w/v crystal violet, solubilised and quantified using an optimal density reading at 595 nm. Results are plotted with standard deviation error bars from triplicate experiments each consisting of eight experimental replicates; the *p* value was calculated using a paired Student's T test. b. The trans-complemented *bpss1439* mutant (pME-*1439*), as well as the wild-type and *bpss1439* pME strain, were also assessed for biofilm production as described previously.

The reported functions of TAAs includes roles in both autoagglutination and biofilm formation [29]. To assess if the unstudied predicted TAA encoded by *bpss1439* has a role in either of these phenotypes, a comparative analysis of the *bpss1439* mutant and its parent wild-type 10276 strain was performed. The strains were incubated for 48 hours in LB at 37°C under static conditions and monitored for altered autoagglutination by the measurement of the optical density of the growth media; the *bpss1439* mutant and wild-type strains demonstrated the same level of autoagglutination (data not shown). However, when these cultures were assessed for their ability to form biofilms, the *bpss1439* mutant demonstrated a significant (*p*<0.001) reduction in biofilm formation at 48 hours in LB at 37°C compared to the wild-type strain (Figure 2a°). This same phenotypic profile was found to occur at temperatures of 27C and 30°C, as well as in minimal media and LB enriched with 50 mM glucose (data not shown).

To confirm that the observed reduction in biofilm formation is due to the disruption of *bpss1439*, rather than downstream genes in the operon (Figure 1), the *bpss1439* mutant was complemented *in trans*. A full-length copy of the *bpss1439*; gene was cloned into the inducible pME6032 vector the resultant plasmid (pME-*1439*) was transformed into the *bpss1439* mutant by electroporation. Additionally, *B. pseudomallei* wild-type and the *bpss1439* strain harbouring empty pME6032 (pME) vector were constructed as controls to account for any potential effects of the plasmid *per se* on biofilm formation. All strains were tested and found to display wild-type levels of *in vitro* growth under the conditions used for the biofilm assays (Figure S1). Biofilm assays were repeated as described previously and the expected levels of biofilm were observed for the wild-type (pME) and *bpss1439* (pME) strains. However, biofilm production by the *bpss1439* (pME-*1439*) strain was restored to wild-type levels in the presence of inducer (Figure 2b). Therefore, it can be concluded that *bpss1439* is a novel factor involved in biofilm formation of *B. pseudomallei*. We have therefore named this gene *bbfA*, *Burkholderia* biofilm factor A.

### The bbfA mutant demonstrated reduced adhesion and microcolony formation

**Figure 3:**
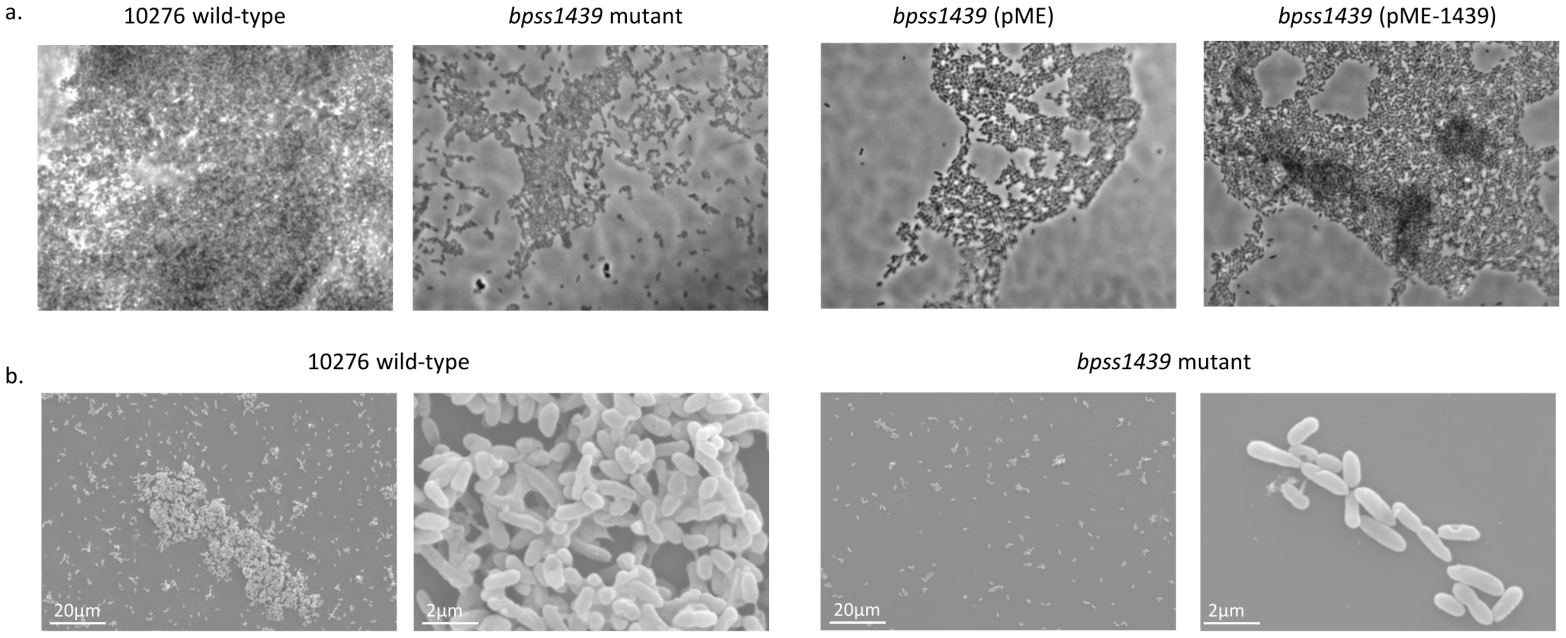
The *bbfA* mutant demonstrated reduced adhesion and microcolony formation. A. The wild-type, *bbfA* mutant and trans-complemented bbfA mutant (pME-*1439*), along with the control wild-type and *bbfA* mutant strains harbouring pME, were grown in LB under static conditions at 37°C for 48 hours. Biofilms were stained for exopolysaccharide using the periodic acid-Schiff protocol and examined by light microscopy. B. The wild-type and *bbfA* mutant biofilms were also fixed with 2.5% v/v glutaldehyde and examined by scanning electron microscopy.

Microscopy was used to confirm and examine the defect in biofilm production by the *bbfA* mutant. The wild-type and *bbfA* mutant, along with the control strains harbouring pME or pME-*1439*, were grown on glass coverslips under static conditions in LB at 37°C for 48 hours. Biofilms were stained for exopolysaccharide using the periodic acid-Schiff protocol. When grown under biofilm-inducing conditions, the *bbfA* mutant demonstrated reduced adhesion as seen by lower total bacterial numbers, as well as reduced microcolony formation as noted by the lack of clumping or autoagglutination of bacterial cells in comparison with the wild-type (Figure 3a). As previously found with the crystal violet biofilm assay, the wild-type (pME) and the complemented *bbfA* (pME-*1439*) strains demonstrated wild-type levels of biofilm production with extensive microcolony formation while the *bbfA* mutant and *bbfA* (pME) strains displayed reduced adhesion and microcolony formation (Figure 3a).

Biofilm formation of the wild-type and *bbfA* strains was further examined by SEM (Figure 3b). These images were congruent with the periodic acid-Schiff staining with the *bbfA* mutant demonstrating reduced adhesion and microcolony production in comparison to the wild-type. Additionally, evidence of biofilm maturation in terms of the depth of the wild-type biofilm formation is clearly lacking in the *bbfA* mutant samples grown in biofilm-inducing conditions. Therefore, *bbfA* appears to play a role in the initial adhesion of *B. pseudomallei* to an abiotic surface based on reduced bacterial numbers at the 48 hour time-point of biofilm formation.

### The bbfA mutant did not demonstrate any of the additional phenotypes reported for other biofilm reduced mutant strains

All previously characterised *B. pseudomallei* mutants with reduced ability to form biofilms also showed pleiotropic effects on multiple other phenotypes. Therefore, to further understand whether the BbfA protein acts directly in biofilm formation, the following phenotypes were examined: polysaccharide capsule (monoclonal antibody staining), LPS (silver stain), flagella and pili (swimming, swarming and twitching motility plates), oxidative stress (peroxide sensitivity), osmotic stress (NaCl sensitivity), dessication (viability post-drying) and exopolysaccharide production (Congo red binding). Additionally, the *bbfA* mutant was tested for net intracellular replication in both epithelial A549 and macrophage J774.2 cell lines. The *bbfA* mutant was found to display wild-type characteristics for all of these phenotypes (Table 2; Figure S2). These data suggest the adhesion phenotype of BbfA-mediated biofilm formation described earlier is directly responsible for the reduced production of biofilm in the *bbfA* mutant.

**Table 2:** Comparison of the *bbfA* mutant with other reported biofilm reduced strains.

Mutant	Annotation	Source	Other phenotypes	Cell lines
*wzn*	ABC transporter	[9]	acapsular, ↓ oxidative survival, desiccation	
*fliC*	Flagellin	[4]	aflagellate	
*cpdA*	c-di-GMP phosphodiesterase	[11]	aflagellate, ↑ EPS†, autoagglutination	↓ A549 & THP1 invasion
*pkk*	polyphosphate kinase	[10]	aflagellate, ↓ oxidative survival	
*rpoE*	Alternative sigma factor	[13]	↓ oxidative & osmotic survival	↓ J774 invasion
*bspI*	QS synthase 1	[12]	no QS 1	
*bspI3*	QS synthase 3	[12]	no QS 3	
*bspR*	QS regulator 1	[12]	no QS 1	
*bbfA*	TAA	This study		wt A549 & J774 invasion

† EPS =  exopolysaccharide.

### The bbfA mutant exhibited an attenuation for virulence in the BALB/c murine melioidosis model compared to the parent strain

**Figure 4:**
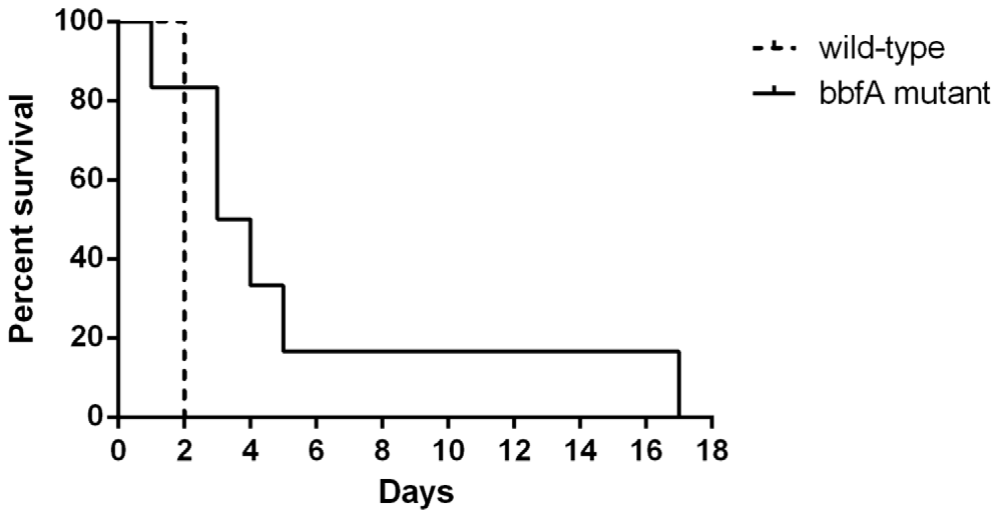
Mice infected with the *bbfA* mutant demonstrated a delay to death in comparison to those challenged with the parental strain. Groups of six BALB/c mice were challenged via the intra-peritoneal route and the median time to death was determined by the Mantel-Cox log-rank test at 35 days post-infection. Mice inoculated with the *bbfA* mutant had a mean survival time of 3.5 days versus 2 days for those dosed with the wild-type (*p* = 0.03).

To assess the role of biofilm formation in the pathogenesis of *B. pseudomallei*, the *bbfA* mutant was tested for virulence in a murine melioidosis model. Groups of six BALB/c mice were challenged via the intra-peritoneal route and median time to death was determined by the Mantel-Cox log-rank test at 35 days post-infection (Figure 4). Compared to mice infected with the parent 10276 wild-type, the mice challenged with the *bbfA* mutant demonstrated a significant delay in time to death, with a mean survival time of 3.5 days versus 2 days for the wild-type (*p* = 0.03). However, this delay in time to death could, in part, be a product of the marginally lower inoculum (3.07×10^5^ CFU for *bbfA* vs. 3.5×10^5^ CFU for the wild-type); therefore, subsequent animal studies were performed. Six groups of five BALB/c mice were challenged via the intra-peritoneal route and the median lethal dose (mice were humanely culled when pre-defined end-points were reached) was determined by the using the Reed and Meunch calculation based on cumulative lethal dose. Compared to the parent 10276 wild-type, the *bbfA* mutant demonstrated a 13-fold increase in medium lethal dose (2.09×10^5^ vs. 1.6×10^4^ CFU). To confirm that this attenuation is directly linked to BbfA, *in trans* complementation of the *bbfA* mutant was performed using a competitive *in vivo* growth assay. An equal ratio of the wild-type harbouring empty pME6032 vector and the *bbfA* mutant harbouring either pME6032 or the pME-*1439* complementation vector was inoculated into groups of three BALB/c mice via the intra-peritoneal route. Mice were humanely culled at 24 hours post-infection and livers and spleens were harvested and plated for bacterial recovery. Colonies were patched to determine the percentage of *in vivo* growth by the *bbfA* mutant versus the wild-type strain by subtracting the number of tet^R^, kan^R^ (*bbfA* mutant harbouring pME6032 or pME-*1439*) colonies from the number of tet^R^, kan^S^ (wild-type harbouring pME6032) colonies. The *bbfA* mutant harbouring empty pME6032 vector demonstrated reduced *in vivo* growth compared to the wild-type harbouring pME6032. However, this attenuation was abrogated by the complemented *bbfA* mutant strain that displayed a comparable level of *in vivo* survival to the wild-type harbouring pME6032, both within the spleen (0.33%±0.58% vs. 42.3%±12.3%, *p* = 0.004) and the liver (0%±0% vs. 42.5%±20.6%, *p* = 0.023). Bacteria isolated from the liver and spleen of control mice infected with only the complemented *bbfA* mutant strain demonstrated between 0% to 4% loss of the pME-*1439* plasmid over the 24 hour experimental period. These data suggest that *bbfA*-mediated biofilm formation may play a subtle role in the pathogenesis of melioidosis. Future studies could focus on whether biofilm production relates to chronic disease rather than the acute infection seen in the BALB/c melioidosis model [6], or when mice are challenged at a mucosal surface rather than by a parenteral route. Alternatively, studies could examine differences between strains; it has been previously observed that *B. pseudomallei* strains demonstrate varying levels of biofilm production, while *B. mallei* and *B. thailandensis* strains produce relatively low levels of biofilm [4], [6], [7]. Therefore, it is of interest to note that the *bbfA* gene occurs as a pseudogene in some *B. pseudomallei* strains, as well as *B. thailandensis* and *B. mallei*, and this may explain the variable biofilm levels seen.

Biofilm formation has been demonstrated to be of importance in dynamic environments where there is a flow of fluid [30]. In agreement with this observation, it has been proposed that the ability of *B. pseudomallei* to form biofilms is important to its colonisation of bore wells where it has been implicated in melioidosis outbreaks [31]. While a role for biofilm formation for persistence in the environment is often referenced [6], [13], [32], it is important to remember that inoculation events, which are essential for pathogenesis, occur at the environmental-host interface. Furthermore, it is tempting to speculate, that similar to the related pathogens *Burkholderia cenocepacia* [33] and *Pseudomonas aeruginosa* [34], autotransporter-mediated biofilm formation of *B. pseudomallei* may be involved in persistent infection of airways and lungs. Unfortunately, little similarity (38% amino acid identity overall) is seen with between BbfA and the *B. cenocepacia* biofilm factor (BCAM0223); the functional domains of TAAs demonstrate significant sequence divergence [35]. Yet an indication for a potential role of biofilm production in the virulence of *B. pseudomallei* is implicated by the identification of *bbfA* as one of the subset of genes within the *B. pseudomallei* core genome which is undergoing positive selection. This subset of genes, which demonstrate an above-background rate of functional variation, is enriched for genes with proposed or experimentally validated roles in virulence [26]. Additionally, B*bfA* was recognised in convalescent sera from melioidosis patients indicating that it is expressed *in vivo* [20]. One could propose that biofilm is important for the unique lifestyle of *B. pseudomallei* in which it transitions between survival, and persistence, in both the environment and various hosts.

## Supporting Information

Figure S1 **The** ***bbfA*** **mutant, and the complemented strains, demonstrate wild-type levels of** ***in vitro*** **growth.** a. The wild-type and *bbfA* mutant were grown in LB at 37°C for 48 hours; *in vitro* growth was quantified using optimal density readings at 600 nm. Average values from biological triplicates are plotted. b. The trans-complemented *bbfA* mutant (pME-*1439*), as well as the wild-type and *bbfA* pME strain, were also assessed for *in vitro* growth under biofilm production conditions. There is a slightly lower final (48 h) OD600 reading for the wild-type strain with pME; this strain demonstrates slightly higher biofilm formation which may affect the OD reading at this time point.
(TIF)

Figure S2 **Phenotypic analysis of the** ***bbfA*** **mutant.** The *bbfA* mutant and its parental wild-type 10276 strain were assessed for various phenotypes which may impact of biofilm formation using published methods. The following phenotypes were examined: a. polysaccharide capsule (monoclonal antibody staining), b. LPS (silver stain), c. flagella and pili (swimming, swarming and twitching motility plates), d. exopolysaccharide production (Congo red binding) and e. intracellular replication in both epithelial A549 and macrophage J774.2 cell lines. The *bbfA* mutant was found to display wild-type characteristics for all of these phenotypes.
(TIF)
