# Correction: Identification of a Predicted Trimeric Autotransporter Adhesin Required for Biofilm Formation of *Burkholderia pseudomallei*

**DOI:** 10.1371/annotation/f016476b-5b84-4c9a-899f-fe8b8bc927b5

**Published:** 2014-01-09

**Authors:** Natalie R. Lazar Adler, Rachel E. Dean, Richard J. Saint, Mark P. Stevens, Joann L. Prior, Timothy P. Atkins, Edouard E. Galyov

An error was introduced in the PDF version of this manuscript during the preparation of this article for publication. Many of the figure references in the Results and Discussion portion do not contain the figure number. 

On page 4, the first sentence of the Results and Discussion should reference Figure 1. The correct sentence is: 

"The B. pseudomallei K96243 genome encodes nine predicted TAAs; the bpss1439 gene is located on chromosome two and has been predicted to be located in an operon with two downstream genes, bpss1442 and bpss1443 (C. Ong, personal communication; [26]) (Figure 1)."

On page 6, there are multiple errors:

1. The first sentence, which continues from page 5, should reference Figure 2a. The correct sentence is: 

"However, when these cultures were assessed for their ability to form biofilms, the bpss1439 mutant demonstrated a significant (p<0.001) reduction in biofilm formation at 48 hours in LB at 37°C compared to the wild-type strain (Figure 2a)."

2. The first sentence in the second paragraph should reference Figure 1. The correct sentence is: 

"To confirm that the observed reduction in biofilm formation is due to the disruption of bpss1439, rather than downstream genes in the operon (Figure 1), the bpss1439 mutant was complemented in trans."

3. The third to last sentence of the third paragraph should reference Figure 2b. The correct sentence is: 

"However, biofilm production by the bpss1439 (pME-1439) strain was restored to wild-type levels in the presence of inducer (Figure 2b)."

4. Both figure references in the first paragraph of the subsection "The bbfA mutant demonstrated reduced adhesion and microcolony formation" should reference Figure 3a. The correct text is:

"When grown under biofilm-inducing conditions, the bbfA mutant demonstrated reduced adhesion as seen by lower total bacterial numbers, as well as reduced microcolony formation as noted by the lack of clumping or autoagglutination of bacterial cells in comparison with the wild-type (Figure 3a). As previously found with the crystal violet biofilm assay, the wild-type (pME) and the complemented bbfA (pME-1439) strains demonstrated wild-type levels of biofilm production with extensive microcolony formation while the bbfA mutant and bbfA (pME) strains displayed reduced adhesion and microcolony formation (Figure 3a)."

5. The first sentence of the second paragraph of the above section should reference Figure 3b. The correct sentence is: "Biofilm formation of the wild-type and bbfA strains was further examined by SEM (Figure 3b)."

6. The second sentence of the subsection "The bbfA mutant exhibited an attenuation for virulence in the BALB/c murine melioidosis model compared to the parent strain: should reference Figure 4. The correct sentence reads: "Groups of six BALB/c mice were challenged via the intra-peritoneal route and median time to death was determined by the Mantel-Cox log-rank test at 35 days post-infection (Figure 4)."

