# Supplementary figures and images for: Identification of a Predicted Trimeric Autotransporter Adhesin Required for Biofilm Formation of Burkholderia pseudomallei
Source: PLoS One. 2013 Nov 5;8(11):e79461. doi: 10.1371/journal.pone.0079461 (PMC3818227; doi:10.1371/journal.pone.0079461)

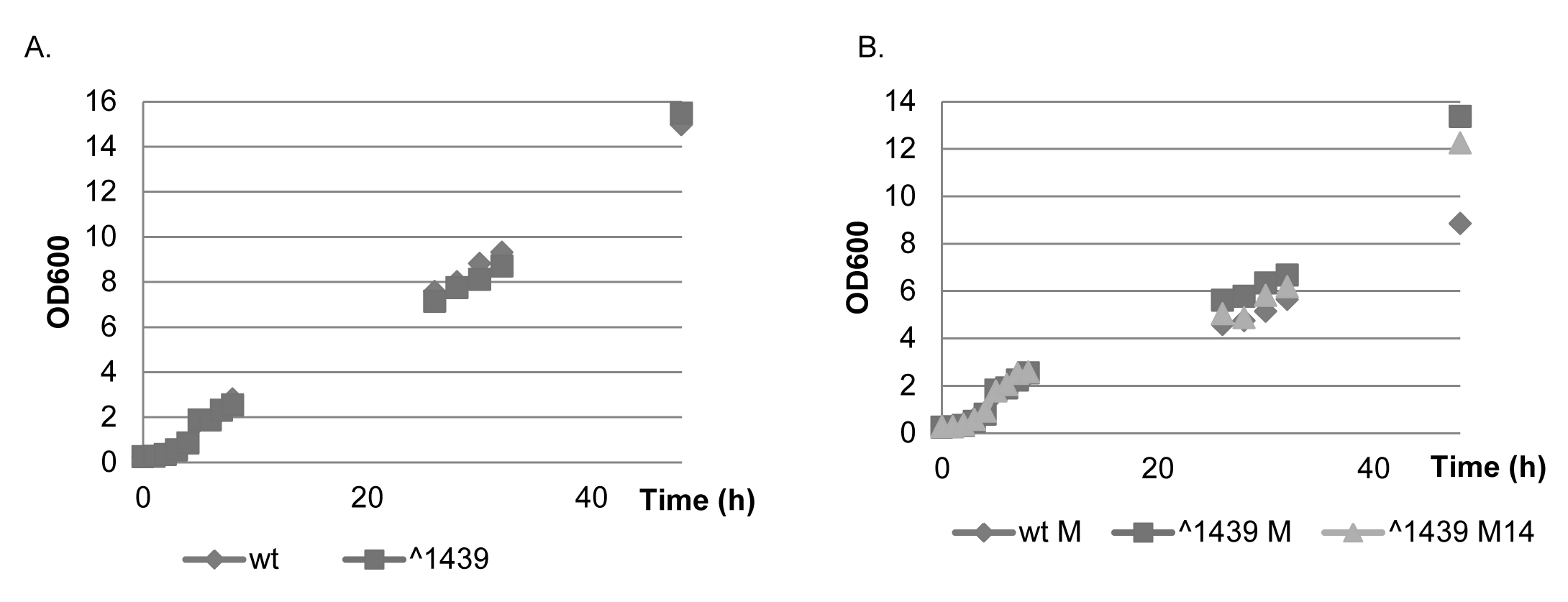

Supplement: Figure S1 — The bbfA mutant, and the complemented strains, demonstrate wild-type levels of in vitro growth. a. The wild-type and bbfA mutant were grown in LB at 37°C for 48 hours; in vitro growth was quantified using optimal density readings at 600 nm. Average values from biological triplicates are plotted. b. The trans-complemented bbfA mutant (pME-1439), as well as the wild-type and bbfA pME strain, were also assessed for in vitro growth under biofilm production conditions. There is a slightly lower final (48 h) OD600 reading for the wild-type strain with pME; this strain demonstrates slightly higher biofilm formation which may affect the OD reading at this time point. (TIF) [file pone.0079461.s001.tif]

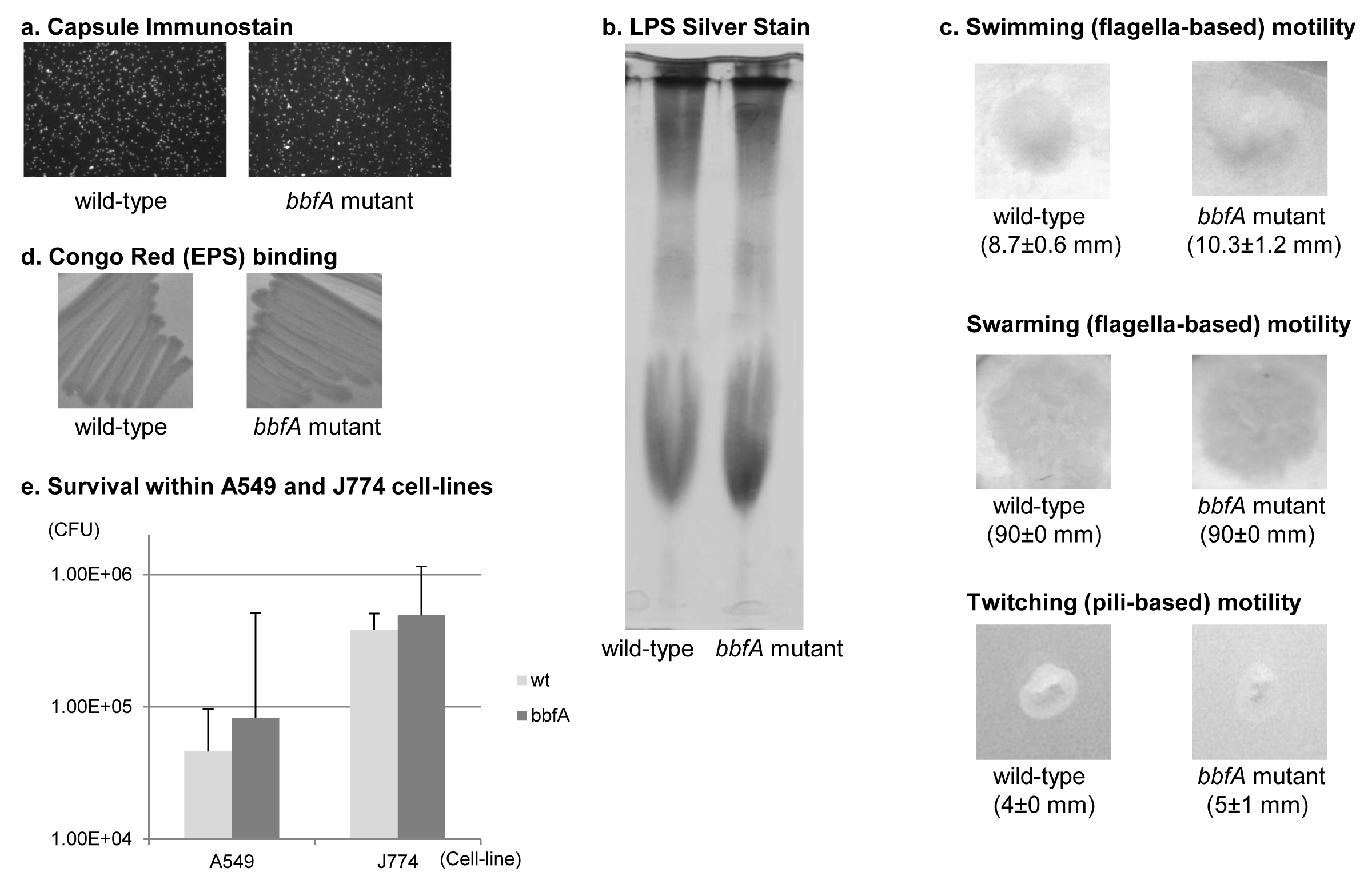

Supplement: Figure S2 — Phenotypic analysis of the bbfA mutant. The bbfA mutant and its parental wild-type 10276 strain were assessed for various phenotypes which may impact of biofilm formation using published methods. The following phenotypes were examined: a. polysaccharide capsule (monoclonal antibody staining), b. LPS (silver stain), c. flagella and pili (swimming, swarming and twitching motility plates), d. exopolysaccharide production (Congo red binding) and e. intracellular replication in both epithelial A549 and macrophage J774.2 cell lines. The bbfA mutant was found to display wild-type characteristics for all of these phenotypes. (TIF) [file pone.0079461.s002.tif]
